# The Pulse of Long COVID on Twitter: A Social Network Analysis

**DOI:** 10.34172/aim.2024.06

**Published:** 2024-01-01

**Authors:** Ikhwan Yuda Kusuma, Suherman Suherman

**Affiliations:** ^1^Institute of Clinical Pharmacy, University of Szeged, H-6725 Szeged, Hungary; ^2^Pharmacy Study Program, Faculty of Health, Universitas Harapan Bangsa, 53182 Purwokerto, Indonesia; ^3^Doctoral School of Educational Sciences, Faculty Humanities and Social Science, University of Szeged, 6722 Szeged, Hungary; ^4^Mathematics Education, Faculty of Teaching and Teacher Education, Universitas Islam Negeri Raden Intan Lampung, Indonesia

**Keywords:** Long COVID, Sentiment analysis, Social network analysis

## Abstract

**Background:**

Long coronavirus disease (COVID) is a complex and multifaceted health condition with a range of severe symptoms that can last for weeks or even months after the acute phase of the illness has passed. Employing social network analysis (SNA) can rapidly provide significant health information to communities related to long COVID. This study aimed to identify the key themes, most influential users, and overall sentiments in the Twitter discourse on long COVID.

**Methods:**

Data were collected from a Twitter search with the specific keywords "long COVID" from December 1, 2022, to February 22, 2023, using NodeXL Pro. Visualizations, including network graphs and key influencers, were created using Gephi, and sentiment analysis was conducted with Azure Machine.

**Results:**

In total, 119,185 tweets from 94325 users were related to long COVID. Top influencers include medical professionals, researchers, journalists, and public figures, with news media platforms as primary information sources; the most common hashtag was #longCOVID, indicating that it is a significant issue of concern among the Twitter community. In the sentiment analysis, most tweets were negative.

**Conclusion:**

The study highlights the importance of critically evaluating information shared by influential users and seeking out multiple sources of information when making health-related decisions. In addition, it emphasizes the value of examining social media conversations to understand public discourse on long COVID and suggests that future researchers could explore the role of social media in shaping public perceptions and behaviors related to health issues. Strategies for enhancing scientific journal engagement and influence in online discussions are discussed as well.

## Introduction

 Long coronavirus disease (COVID), also known as post-acute sequelae of severe acute respiratory syndrome coronavirus 2 infection, is a multisystemic health condition characterized by a range of severe symptoms that develop following the primary infection.^1^ This multisystemic illness encompasses myalgic encephalomyelitis/chronic fatigue syndrome (ME/CFS), dysautonomia, impacts on the metabolic system (e.g., diabetes mellitus,^2^ the cardiovascular system,^3^ and the nervous system such as ME/CFS)^4,5^ among other systems, as well as vascular and clotting abnormalities.^4^ According to a conservative estimate, at least 65 million individuals worldwide are currently affected by long COVID, based on a documented incidence of 10% among over 651 million COVID-19 cases globally^1^; however, this number could be much higher because many cases have not been documented yet.

 Long COVID is characterized by a range of symptoms that persist for weeks or even months after the acute phase of the illness has passed,^6^ with common symptoms such as fatigue, shortness of breath, chest pain, brain fog, memory problems, sleep disturbances, anxiety, depression, and joint pain.^7,8^ Although most people who contract COVID-19 experience mild to moderate symptoms and recover fully within a few weeks, some continue to experience symptoms for an extended period, often without clear resolution.^4,7^ Specific prevalences of long COVID vary, with estimates ranging from 10% to 30% of non-hospitalized cases, 50%–70% of hospitalized cases,^9,10^ and 10%–12% of vaccinated cases.^11^ However, the increasing number of long COVID cases necessitates further research to better understand this condition and develop effective management strategies given the substantial impact it can have on the quality of life of affected individuals, including their ability to perform daily activities such as returning to work.^12^ Social media platforms, particularly Twitter, can rapidly provide significant health information to communities, including information on long COVID.^13^

 Social network analysis (SNA) is a powerful tool for visualizing complex social networks on social media platforms such as Twitter.^14^ In recent years, SNA has been increasingly used in public health research, including research on infectious diseases such as COVID-19,^15,16^ Zika,^17^ and Ebola.^18^ However, there has been limited research on using SNA to study long COVID. The researchers of recent studies who utilized SNA only focused on conversations using specific hashtags^19^ or only employed sentiment analysis.^20-22^

 Our aim with this study was to explore the long COVID conversation on Twitter, and SNA has been applied to identify the key themes and patterns in large datasets, such as those from social media platforms, the most influential users in networks, and the overall user sentiment of the conversation.^23,24^ In this study, the analyses were performed using NodeXL Professional (version 1.0.1.508, Social Media Research Foundation, Redwood City, CA, USA), a user-friendly SNA tool. NodeXL’s data visualization capabilities were used to create clear and informative graphic displays of the long COVID conversation on Twitter. We aimed to provide a deeper understanding of the long COVID experience that could inform efforts to support affected individuals in managing the condition through new interventions and to raise awareness and understanding of the condition among the wider public.

## Data Collection

NodeXL Pro software enables collecting tweets from Twitter.^25^ Thus, it was utilized to search for tweets that contained specific keywords such as “long COVID”. The data were collected from December 1, 2022, to February 22, 2023. The collected tweets consisted of text, user information (e.g., username, location, and number of followers), and metadata (e.g., user ID, tweet ID, and time of posting). Finally, the tweet IDs were entered into NodeXL as edges. In addition, Microsoft Excel 2019 was employed to retrieve data from NodeXL. NodeXL uses Twitter’s search network interface, and URLs were automatically expanded within NodeXL.

###  Data Cleaning

The data cleaning process included eliminating duplicate and irrelevant tweets from the collected data. Then, the data were pre-processed to remove stop words, punctuation, and special characters from the tweets’ content. The user data and metadata were used to develop a user-tweet network in which people were represented as nodes and tweets as edges.^26^

###  Data Analysis

SNA was utilized to examine the social structures of Twitter conversations about long COVID by analyzing their relationships.

####  Network Graph

Gephi software (Version 0.1.0.), a network visualization software,^27^ was employed to create a network graph of the long COVID conversation on Twitter from the NodeXL vertex output. The nodes in the graph represent individual Twitter users, and the edges represent the relationships between them based on retweets, mentions, and replies. NodeXL used built-in measures of network centrality to identify the most influential nodes in the network, that is, those with high betweenness centrality; betweenness centrality represents the number of shortest paths that pass through a given node in the network. The size of the nodes demonstrates the number of followers, and the colour of the nodes represents the sentiment of the tweets. The YifanHu multilevel layout in Gephi software was employed to visualize the edge networks.^28^

####  Top Influencer

The top influencer could be identified as the node (user) with the highest betweenness centrality. Nodes with a high betweenness centrality have a central role in connecting different network parts.^29^

####  Top Topics and Sources

Identifying the topics and patterns of conversation on Twitter can provide valuable insights into users’ interests, preferences, and concerns; the top URLs, domains/sources, and hashtags related to long COVID were analyzed for this study. Key themes and sources were primarily identified from the URLs and domains/sources related to long COVID that people shared most often. However, hashtags are important features of Twitter that are used to categorize tweets and make them easier to find, and analyzing the top hashtags related to long COVID also provided insights into the key topics and themes discussed on Twitter.

####  Sentiment Analysis

Sentiment analysis involves analyzing text to identify the emotional tone and polarity of the language used; the technique was utilized to classify Twitter users’ opinions of long COVID as positive, negative, or neutral. Our results were confirmed using Azure Machine Learning to separately calculate tweet sentiment scores.^30^ A tweet that expressed hope and optimism about a new treatment for long COVID was classified as positive; in contrast, a tweet that expressed frustration with the lack of progress in finding a cure was categorized as negative, and a tweet that just shared information about long COVID without emotion was classified as neutral. The sentiment analysis output can be used to track changes in public sentiment about long COVID over time and identify any emerging trends or issues that need further investigation.

## Results

**Figure 1:**
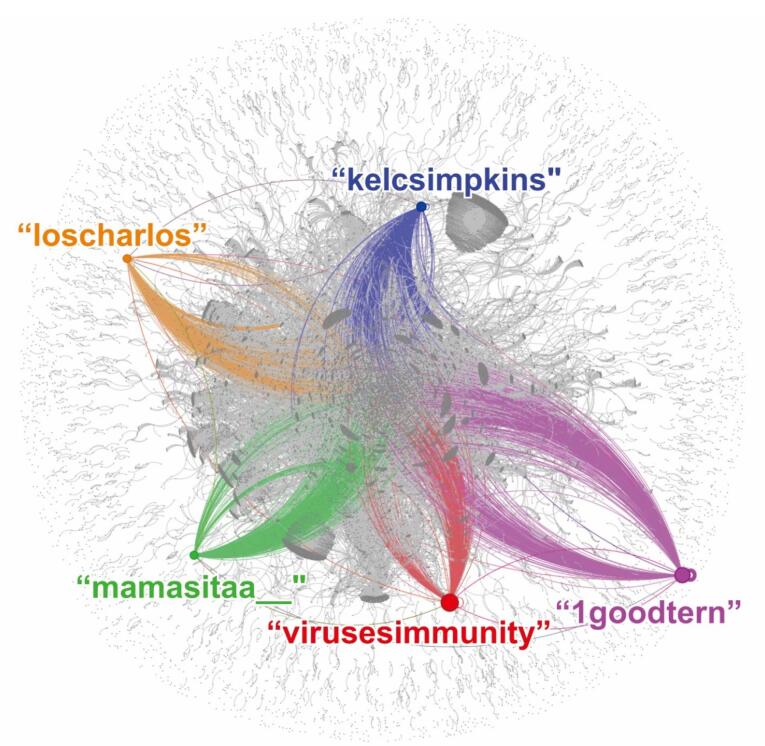
The Social Network Map

 The SNA of long COVID-related tweets collected between December 1, 2022, and February 22, 2023, yielded a total of119 185 tweets from 94 325 users. Figure 1 illustrates the network graph of the betweenness centrality for the top five Twitter users, which provides insights into the structure and organization of the network and identifies which users are playing a key role in connecting different parts of the network.

 As discussed, betweenness centrality measures how often a user serves as a bridge between other users or groups of users. The results showed that the user @virusesimmunity (red node) had the highest betweenness centrality, implying that they were the most important users in the network in terms of bridging connections between other users. The subsequent four users with the highest betweenness centrality were @1goodtern (purple node), @kelcsimpkins (blue node), @mamasitaa__ (green node), and @loscharlos (orange node) in descending order. This information could guide targeted interventions or communication strategies to address long COVID issues on Twitter.

###  Top Influencer

**Figure 2:**
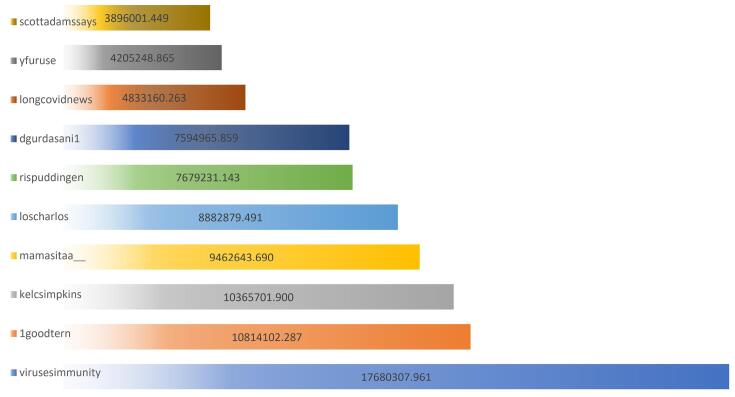
The Top Betweenness Centrality Users (The Most Influencers)

Figure 2 displays the actual betweenness centrality values for the top ten users regarding the topic of long COVID. Again, the user “virusesimmunity” was the most influential, with a betweenness centrality of 17680307.961; this user appeared to be a leading expert in virology and immunology, as evidenced by their Twitter handle. The second most influential user was “1goodtern” with a betweenness centrality of 10814102.287; this user appeared to be a medical doctor actively sharing information about long COVID. The user “kelcsimpkins” was a freelance journalist sharing news articles and scientific research on long COVID, and the other highly influential users represented a mix of medical professionals, researchers, journalists, and public figures actively discussing long COVID on Twitter.

###  Top Topics and Sources

**Figure 3:**
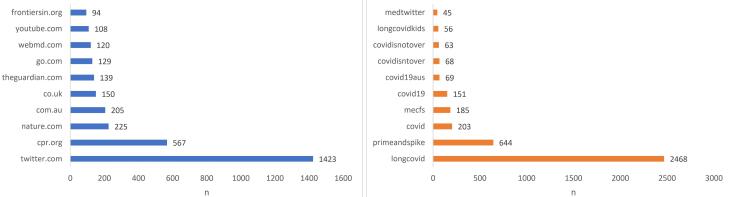
The Most Domain/Source **(a)** and the Most Hashtags in Tweets **(b)**

Based on the analysis results (Figure 3), the most shared URL was from CPR News, with 562 shares. This was followed by a post by Dr. Tad, a physician, with 173 shares, and an article from WebMD News, with 116 shares (Table 1). Regarding user categories, news media accounts—specifically those for CPR News, WebMD News, ABC News, the Tyee, Nursing Times, and the Sydney Morning Herald—shared most of the top URLs related to long COVID. Science journals, including *Nature Review* and *Frontiers in Immunology*, were also among the top sources of information. It is worth noting that the findings suggest a significant interest in long COVID among users on Twitter, particularly about the potential causes and treatments of the condition. The high number of shares for articles related to long COVID suggests that this topic is of great concern and interest among both medical professionals and the general public.

**Table 1:** The Most Topics and Sources Discussed Based on the Most URL

**User Category**	**Source**	**Title/Content**	**Number**
News Media	CPR News	As many as 1 in 10 Coloradans may have been hit by long COVID, a report says	562
Physician	Dr. Tad	“There are data showing the seriousness of the after-effects of infection with the Omicron strain. According to a survey conducted by Okayama University Hospital, sleep disorders and fatigue increased significantly compared to the Delta strain.” A specialist mentioned, “Continue infection control without letting your guard down, even after shifting to the same type 5 as seasonal influenza.”	173
News Media	WebMD News	Inflammation and Immunity Troubles of the Top Long COVID Suspect List	116
News Media	ABC News	I felt powerless’: Black Americans suffering from long COVID indicate that they have trouble accessing care	104
Scientific journal	Nature Review	Long COVID: Major Findings, Mechanisms, and Recommendations	103
Health insurance	Value Penguin	Nearly 36 Million Americans Who Tested Positive for COVID-19 Reports Having Long COVID Symptoms — Including More Than 40% in Mississippi	91
News Media	The Tyee	Long COVID Has Never Been Taken Seriously. Here’s Where It Left Us	86
News Media	Nursing Times	An exclusive survey reveals ‘worryingly high’ levels of long COVID among nurses	80
News Media	The Sydney Morning Herald	Long COVID clinics and patients in limbo after federal funding expires	68
Scientific journal	Frontiers in Immunology	Long-term high-dose immunoglobulin successfully treats long COVID patients with pulmonary, neurologic, and cardiologic symptoms	65

Figure 3a shows the top domains in the collected tweets, revealing that Twitter was the most common source, accounting for 1423 out of 5000 tweets. This is not surprising given that Twitter is a social media platform and a popular place for users to share and discuss information about various topics. Figure 3b depicts the top hashtags in the tweets; notably, long COVID, with 2468 mentions, was the most frequently discussed topic among Twitter users related to COVID-19. It is worth noting that the second most commonly used hashtag was #primeandspike, with 644 mentions. #Primeandspike refers to a Twitter conversation initiated by Dr. Eric Feigl-Ding, an epidemiologist, in which he shared data about the highly transmissible omicron variant and called for increased caution in response to it. This hashtag demonstrates that Twitter users are actively engaged in discussing and sharing information about emerging developments in the COVID-19 pandemic.

Figure 3b illustrates the other most notable hashtags, and we highlight that the hashtag #COVIDisntover (68 mentions) suggests that Twitter users are still actively discussing and raising awareness about the ongoing impact of COVID-19. Interestingly, #mecfs was among the most common hashtags because many people with long COVID experience similar symptoms to those of ME/CFS, such as fatigue, pain, cognitive dysfunction, and post-exertional malaise. The tag #longCOVIDkids (56 mentions) highlighted the particular concerns of parents and caregivers for children who are experiencing long COVID symptoms.

###  Sentiment Analysis

**Figure 4:**
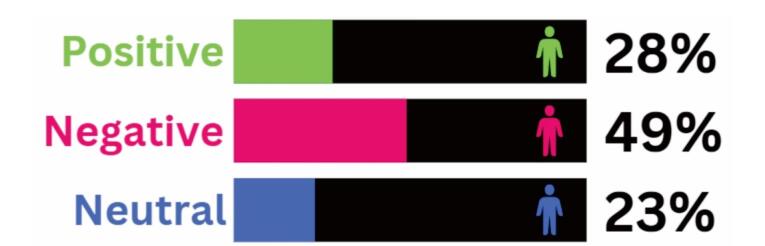
Sentiment Analysis Related to Long COVID

Sentiment analysis using Azure Machine Learning indicated that most of the tweets we collected (49%) were negative, suggesting significant concern, frustration, and possibly fear associated with long COVID (Figure 4). Only 28% of the tweets reflected a positive sentiment, which could demonstrate that there is still much uncertainty around the long-term effects of COVID-19 and/or that many people are still struggling with the ongoing impact of the disease. The final 23% of the tweets were neutral, simply presenting factual information, personal experiences, and opinion pieces, for example. Overall, the sentiment analysis results provide insight into the emotions and opinions of Twitter users related to long COVID, which could help in understanding public perceptions and attitudes toward the disease.

## Discussion

 The use of SNA to analyze long COVID-related tweets provided valuable insights into the structure and organization of the Twitter networks that have developed on the topic of long COVID; it allows for identifying which users play a key role in connecting different network parts related to the subject. The betweenness centrality of a given user is a useful measure of that user’s importance in the network based on the number of shortest paths that go through that user’s node. It was found that the user @virusesimmunity had the highest betweenness centrality and therefore was the most important user in the network regarding bridging connections between other users on the subject of long COVID.

 Previous researchers have also used SNA to understand the structure and organization of online communities related to other health issues. For example, Olszowski et al^31^ employed SNA to analyze the structure of online communities related to COVID-19 vaccination and found that the majority of tweets in the network were against mandatory vaccination; the most influential users in the network were primarily antivaccination advocates and conspiracy theorists. They also demonstrated the usefulness of SNA in understanding the structure and organization of online discussions related to public health issues and provided a valuable method for monitoring and addressing public health concerns on social media platforms such as Twitter.

 Similarly, Ahmed and Lugovic^32^ employed SNA to analyze the online conversations on Twitter regarding the relationship between blood clots and vaccines and to identify influential users and communities in the discussion and concluded that the discussion was highly polarized, with different groups expressing opposing views and sharing different types of information. Interestingly, the researchers also found that users with the most followers were not necessarily the most influential, highlighting the importance of network centrality and connectivity in determining influence.^32^ Overall, SNA allows for examining social media conversations to understand public discourse around important social issues.^33^

 The results from this study have provided significant insights into the most influential users on Twitter discussing long COVID, who likely have substantial impacts on shaping public opinion and guiding discussions on the topic. For example, “virusesimmunity,” the most influential user, is an expert in virology and immunology, which makes them a highly influential and reliable source of information; the second most influential user, “1goodtern,” is a medical doctor, which adds to their credibility and influence. The mix of backgrounds among the influential users indicates that a diverse group of people are discussing long COVID on Twitter with different perspectives based on their experiences and expertise; nonetheless, their common goal of sharing information and raising awareness about long COVID is evident. This study’s findings align with those of previous research showing that influential social media users can shape public opinion and behavior.

 Moreover, media Twitter accounts play a critical role in molding public discourse and raising awareness about health concerns.^34^ For instance, Kydros et al^35^ found that influential users on Twitter disseminated accurate information about COVID-19 during the early stages of the pandemic, and as discussed, the high betweenness centrality of “virusesimmunity” indicates that they were a crucial connector between different users in the network discussing long COVID on Twitter. However, high betweenness centrality does not necessarily demonstrate the accuracy or quality of the information a user shares. Therefore, it is crucial to critically evaluate the information being shared by highly influential users and to consider multiple sources of information when making decisions about health and medical issues.

 Moreover, news media accounts shared most of the top URLs related to long COVID, for what could be multiple reasons. One possibility is that news outlets have recognized the significant public interest in long COVID and are actively seeking and sharing related information. Long COVID is a relatively new and rapidly evolving area of research, so there might still be limited information available in the scientific literature. News outlets could be filling this gap by gathering and disseminating information on long COVID from various sources, such as science journals, medical professionals, and patient advocacy groups. Another possibility is that news outlets share information on long COVID as part of their broader coverage of the COVID-19 pandemic. Long COVID is a potential complication of COVID-19, and as such, it is closely linked to the overall impact of the pandemic; news outlets could be sharing information on long COVID to provide a complete picture of the pandemic’s effects on individuals and communities.

 Finally, it is also possible that news outlets are sharing information on long COVID to attract and retain audiences. The COVID-19 pandemic has been a major news story for over two years, and news outlets could be seeking to keep audiences engaged by providing coverage of related topics such as long COVID. Sharing information on long COVID could also be a way for news media outlets to position themselves as trusted sources of information on health and medical issues.

 Regarding the most discussed topics and sources based on the highest number of URLs, it was found that news media accounts were the most active users, with CPR News having the highest number of shares at 562; in contrast, science journals were the least active category, with only 65 shares. A potential explanation for the news media’s high number of shared URLs related to long COVID is their tendency to prioritize topics that are of interest to their audiences and are relevant to current events. For instance, long COVID was the first illness to emerge through patients connecting on Twitter.^36^ Therefore, news accounts might be more likely to cover this topic in their reporting and share articles related to long COVID with their audience.^37^

 In contrast, science journals typically have a more specialized audience and tend to focus more on publishing research findings than on providing news updates on specific topics. Scientific research on long COVID is ongoing,^38,39^ with many challenges,^40^ and there might not be as many published articles on the topic as there are news articles. However, it is important to note that news media and science journals are both important in informing the public about health topics such as long COVID. The news media can help raise awareness about the condition and provide updates on new developments,^41^ and journals can provide more in-depth analysis of the underlying causes and potential treatments. Ultimately, a combination of both news media and scientific research is needed to provide a comprehensive understanding of long COVID, inform the public and medical professionals about this emerging health issue, and raise awareness of the condition among healthcare professionals and the public.^8^

 Furthermore, the most frequently discussed topic on Twitter related to COVID-19 was long COVID, indicating that it is a significant issue of concern among the Twitter community. The mention of the #primeandspike hashtag in the study is significant because it shows that Dr. Eric Feigl-Ding’s Twitter conversation significantly impacted the conversation surrounding vaccines and the COVID-19 pandemic. The fact that it was the second most commonly used hashtag in the data set represents that many Twitter users were engaging with and discussing the topic, which could have had an impact on public perceptions and attitudes toward vaccines. This highlights the potential for social media platforms such as Twitter to shape public discourse and influence public health outcomes.

 Additionally, the high use of the hashtag #mecfs reflects that long COVID shares similarities with ME/CFS, highlighting the importance of understanding and addressing the symptoms and impacts of long COVID.^42^ Additionally, the hashtag #longcovidkids emphasizes the particular concerns of parents and caregivers for children experiencing long COVID symptoms. However, in the context of COVID-19, observing hashtags promoted by health organizations is relevant to recommending more effective campaigns related to topics.^43^

 The high percentage of negative sentiments expressed in tweets related to long COVID, as identified by sentiment analysis using Azure Machine Learning, suggests significant concern and frustration among individuals affected by this condition. This negative sentiment could be attributed to various factors, including the novelty of long COVID and the lack of understanding surrounding it.^44^ Additionally, individuals with long COVID often report ongoing symptoms and a reduced quality of life, which can contribute to negative emotions and frustration.^45^ Finally, the ongoing pandemic has caused significant disruption and stress in daily life, leading to fatigue and burnout in many individuals.^46^ These factors, combined with the uncertainty surrounding long COVID, could contribute to the high percentage of negative sentiment expressed in the tweets.

 In contrast, positive sentiments only account for 28% of the tweets related to long COVID, suggesting that there might still be much uncertainty and concern surrounding the disease. When people are struggling with a condition that is not well understood and has ongoing effects on their health and quality of life, it can be difficult to maintain a positive outlook.^47^ Additionally, discussions around long COVID can include personal experiences and opinion pieces, which do not always have a positive tone.^48^ This lack of positivity in discussions related to long COVID could lead to a sense of hopelessness and a lack of optimism among individuals affected by the disease.

 Finally, neutral sentiments (23%) in discussions related to long COVID could be attributed to the nature of the topic, which is complex and multifaceted. Discussions around long COVID can include factual information, personal experiences, and opinion pieces that do not necessarily elicit strong emotions (e.g., tweets that just provide information on the symptoms and management of long COVID).^49^ Similarly, tweets that express personal experiences with long COVID might not necessarily express a strong sentiment, as individuals can have varying perspectives and attitudes toward their condition.^50^ Thus, the neutral sentiments observed in the sentiment analysis reflect the varied and nuanced nature of the discussions related to long COVID on Twitter.

## Conclusion

 SNA demonstrates that @virusesimmunity and @1goodtern were the most influential users in long COVID discussions on Twitter and that news media accounts, particularly CPR News, were the most active users. News outlets appear to play a significant role in amplifying and shaping discussions on social media platforms, including discussions about health issues, which can affect how the public perceives and responds to different issues. Interestingly, the science journals had the least active Twitter users. Given the importance of scientific research in shaping our understanding of long COVID, it is crucial to explore why scientific journals are not more actively engaged in Twitter discussions on long COVID.

 In light of these findings, it is essential for social media users to critically evaluate the information being shared by highly influential users and to seek out multiple sources of information when making decisions about health and medical issues. This study underscores the value of examining social media conversations to understand public discourse around important social issues such as long COVID. Future researchers could explore the role of other social media platforms in shaping public perceptions and behaviors related to health issues as well as strategies to increase the engagement of science journals in Twitter discussions and their influences. The author reports no conflict of interests in this work.
